# One stop or full stop? The continuing challenges for researchers despite the new streamlined NHS research governance process

**DOI:** 10.1186/1472-6963-10-124

**Published:** 2010-05-13

**Authors:** Andrew GH Thompson, Emma F France

**Affiliations:** 1 Politics and International Relations, School of Social and Political Science, University of Edinburgh, Chrystal Macmillan Building, 15A George Square, Edinburgh EH8 9LD, UK; 2 Department of Nursing & Midwifery, University of Stirling, R.G. Bomont Building, Stirling FK9 4LA, UK

## Abstract

**Background:**

Obtaining the necessary approvals and permission for clinical research requires successful negotiation of the ethical and R&D layers of the NHS. Differences in structure and governance frameworks feature between the constituent nations of the UK (England, Scotland, Wales and Northern Ireland), which adds complexity to cross-national studies. Difficulties in carrying out research in the NHS in the UK due to bureaucratic and time-consuming governance processes have led to the development of a new system of application and co-ordination from 2009. This paper illustrates how this new system fails to be consistent and streamlined and is unlikely to become so unless changes are made to the implementation and management of the governance processes.

**Methods:**

We present a case study of the research governance process at the survey stage of an investigation into the use, preferences and need for information by people making choices or decisions about health care. The method involved home-based, face-to-face interviewing in a questionnaire survey in relation to decisions about lymphoma treatment, Down's syndrome screening in pregnancy, and caring for people with dementia.

**Results:**

Our experience of the ethics stage was very positive, noting an efficient process of application and a speedy decision, both in relation to the initial application and to subsequent substantial amendments. By contrast, the R&D stages were very slow, most with unexplained delays, but some offering contradictory advice and exhibiting a lack of clear guidance and training for NHS staff. The R&D arrangements in Scotland were far quicker and more likely to be successful than in England. Overall, the delays were so severe that substantial parts of the research could not be delivered as planned within the funding timescale.

**Conclusions:**

If high-quality research in the NHS, particularly in England, is to be delivered in a timely and cost-effective way, R&D processes for gaining research governance approval need improvement. Attention is needed in process implementation and management, particularly in relation to staff training, as well as clarity in guidance and communication within and between organisations.

## Background

### A new system providing a consistent and streamlined process for gaining NHS permission for clinical research in England

Against a backdrop of severe and sustained criticism of the NHS research governance arrangements in the UK over the last 10 years, the above strapline [1] heralded a more efficient and integrated system for conducting research in the NHS from 2009 onwards. This paper illustrates how this new system fails to be consistent and streamlined, and is unlikely to become so unless a number of concomitant changes are made to the implementation and management of the governance processes.

### Ethical approval

Ethical practice in research can be reasonably expected by the public to be part and parcel of the normative professional code of conduct. Nonetheless, increasing external regulation, emanating from the Helsinki Declaration in 1964 [2] onwards as a result of notorious aberrations in trust, has moved ethical practice from agency-based to institutional-based governance. Health services research in the UK has epitomised this trend. Parallel developments in the NHS and research-based organisations, such as universities and professional organisations (e.g. Social Research Association; British Sociological Association; Market Research Society), have led to numerous ethical guidelines and frameworks to which professional researchers are expected to adhere. It is now commonplace for universities to require research proposals of any kind, including student projects, to be submitted to an internal ethics committee prior to fieldwork.

Researchers' frustrations, concerns and complaints about excessive time delays in carrying out investigations within the NHS, both clinical [3-5] and social [6-8], are well-documented. Despite the advantages that the institutions of governance and regulation bestow upon investigators by giving licence to their activities, this does not mean that such criticisms are over-stated, as mere "whining of a privileged community" [9]. Researchers have multiple roles to play, not simply as people skilled in the investigatory process, but as advocates for their research participants in giving voice to often unheard social groups, as drivers of quality improvement through timely publication of new knowledge, and, in the case of non-commercial funding, as guardians of the public purse through efficient organisation and management.

Similar developments are taking place elsewhere in the world, at a varying pace, but in the same direction, with related concerns about the efficacy of the governance arrangements. There are major differences between European countries as to what needs to be submitted to Research Ethics Committees (RECs), with the UK being noted as having an arduous process [10]. In the USA federal policies on human subjects research has become strongly protectionist [11], with increased strictures on governing research by the Institutional Review Boards [12]. Problems in the under-resourcing and overworking, not to mention the lack of clear accountability structures, of Human Research Ethics Committees in Australia [13] and Research Ethics Boards in Canada [14], have led to demands to reform their governance systems.

The process of gaining access via the NHS to carry out research with any person or group, be they staff, patients, patient representatives, or even members of the public sampled via NHS records (such as unpaid carers), involves several stages of approval and permission. Before anything else is the requirement to obtain ethical approval, which, despite its bureaucratic and time-consuming nature [8], has shown welcome signs of improvement as a result of cumulative operational changes. Multi-centre investigations, which previously required permission from each Local Research Ethics Committee (LREC), each with its own idiosyncratic systems and viewpoints [15], as well as a global view by the Multi-Centre Research Ethics Committee (MREC), evolved by 2000 into a more co-ordinated system under the Central Office for Research Ethics Committees (COREC), with performance deadlines added subsequently. McDonach et al's study in Scotland [16] reported a delay of only ten weeks between application and approval by the MREC, albeit requiring a further seven weeks for LRECs to confirm their notification of the study, while van Teijlingen et al's study [8] received approval from the MREC within six weeks, but was delayed by another three and a half months awaiting endorsement by the LRECs.

In 2007 COREC was incorporated into the National Research Ethics Service (NRES) of the National Patient Safety Agency, which in turn developed the Integrated Research Application System (IRAS) in 2008 (and fully operationalised in 2009) in response to the need for "a single online system for applying for permissions and approvals for health and social care/community research in the UK" [17]. For researchers working across administrative and, importantly, national boundaries within the UK, given the devolved responsibility for the NHS, this is potentially of great benefit.

### R&D management approval

Having successfully been approved by the ethics reviewing bodies, whose role is to ensure that the potential benefits of research do not exploit or cause harm to vulnerable participants [18], the next stage requires obtaining Research Management and Governance (RM&G) approval, or R&D approval as it is commonly known, from each of the NHS service providers, to ensure they have adequate arrangements and resources in place to ensure quality of care, patient safety and finance to cover staff time [19]. At this point the differences between the nations emerge due to different NHS structures and related policies on governance. In England this involves up to 8 types of Trust, covering more than 400 organisations [20], depending on whether the research participants are to be sampled in primary, secondary, or community services. By contrast, in Scotland it requires application to any of 14 Health Boards, which work across all services in their geographical area. Previously multi-centre studies required application to each Trust or Board individually, whereas under the new system one coordinating centre is vested with the responsibility for leading on all approvals wherever they are required in the UK.

In 2006 the Department of Health developed a new research framework for the NHS in England [21], to create more cohesion between the different organisations and funding streams and to strengthen a competitive research framework in order to increase research investment in clinical trials by the pharmaceutical industry. The fragmentation of the English NHS incurs greater challenges in achieving permission in multi-centre studies, for which the National Institute for Health Research (NIHR), the new research arm of the NHS, has instituted the Coordinated System for gaining NHS Permission (NIHR CSP) to offer a 'one-stop shop' for approvals, thereby aiming to reduce the bureaucratic burden and the time delays that have characterised the process [22]. This system is currently only accessed by studies in the NIHR Clinical Research Network Portfolio via IRAS.

**Figure 1:**
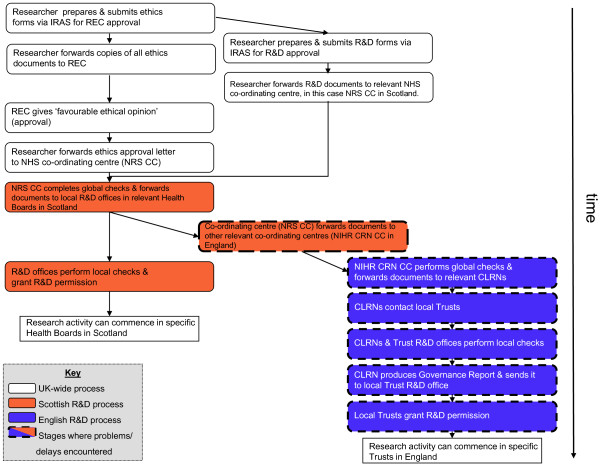
**The new NHS research governance processes in England and Scotland**.

The responsibility for developing the NIHR CSP is vested in the NIHR Clinical Research Network Coordinating Centre (NIHR CRN CC), which undertakes global checks; these are study-wide governance checks which apply to all participating Trusts and that are only carried out once for each study, such as ensuring ethical approval is in place. The Coordinating Centre then asks the relevant Comprehensive Local Research Networks (CLRN), of which there are 25, to undertake local governance checks on behalf of each participating service provider in their area, before forwarding a Governance Report to them [23]. After these approvals have been gained, it is then the responsibility of the local Trusts to give permission through their R&D offices, either by making site-specific assessments (SSAs) of the suitability of the NHS research site and of local researchers in the case where NHS staff are involved in performing the research, or by approving the provider as a Participant Identification Centre (PIC) when their staff are only involved in helping a remote research team with the sampling of participants. Given the multiplicity of layers for gaining permission to carry out research, the effectiveness of the new coordinated system in England is crucial to its success. The comparatively simpler structure in Scotland was found by McDonach et al [16] to require up to 33 additional weeks to gain approvals from the 26 R&D departments involved in their national study. The new structures for the research governance processes in England and Scotland are shown in Figure 1.

The implementation and failures of the latest system that was designed to reduce bureaucracy and time delays in research [24] can be well illustrated by a case study based on our investigation. Our study was one of the first to access the new IRAS application system and so provides an opportunity to test its viability and proclaimed advantages.

## Methods

Through a competitive tendering process, we secured a contract with the NIHR Service Delivery and Organisation to investigate the use, preferences and need for information by patients and unpaid carers to make choices about health care [25]. The research design involved three distinct stages that iteratively employed a variety of methods to build a detailed and nuanced understanding of what and how people talked about information in relation to five specific conditions. Since the first stage involved secondary data analysis that had pre-existing ethical approval, we decided to seek ethical permission for the next two stages in two corresponding phases, the first of which preceded the most recent application process via IRAS.

The case study we describe below concerns the final stage of this design, which involves a questionnaire survey in Scotland and England, using face-to-face, home-based interviews, of people who faced decisions in one of three situations: whether to have treatment for a diagnosis of lymphoma; whether to have an antenatal test for Down's syndrome; and, where a person with dementia should live by people caring for them in an unpaid capacity (e.g. at their home, or in a residential or nursing care home). Only those in the first of these groups would be likely to be patients at the time of the study, but we wished to sample all groups via NHS records in order to allow a high chance of locating such people. The intention was to make generalisable estimates about the distribution of experiences and opinions between and across all groups and both nations. This necessitated random samples being drawn on a clustered geographical basis from secondary care lymphoma clinics and from general practices for the two other groups. Invitations were to be issued by the hospitals and general practices to prospective participants on our behalf. Although reflecting on past decisions could raise respondent anxiety, the risks to participants in such a study are believed to be very small.

The fieldwork was to be sub-contracted to a commercial survey organisation due to the size of the sample necessary to produce generalisable results with a high degree of precision, estimated at 900 in total, coupled with the need to be able to interview people in their homes over a relatively short period in multiple locations across Scotland and England. Any delays would have implications for the costs of the study, since protracted fieldwork and uncertainties about gaining permission would add significantly to the unanticipated costs of securing interviewers in the field, as well as the administration costs of extending contract deadlines.

## Results

### Ethical approval

Due to the multi-centre nature of this research stage, ethical approval was sought via one of the Research Ethics Committees (RECs) in Scotland, which acted on behalf of all RECs. Despite a decision being expected within the obligatory maximum of 60 days, initial approval was secured, with no amendments required, in only 18 working days. Three subsequent 'substantial amendments' were submitted for approval, two relating to revisions to the questionnaire and one a revision of the recruitment process. These three amendments again received favourable opinion quickly, in 11, 12 and 18 working days, well within the obligatory 35 day limit. Under the new system, once the main REC has given approval, there is no longer a need to wait for the RECs in the other sites to approve or acknowledge the ethical nature of the study, since this is communicated via IRAS. Our experience of the ethical review stage was very positive.

### R&D management approval in Scotland

Research management and governance approval, the next step in the process, proved to be a considerable impediment to the successful execution of the project. A local NHS advisor's confusion about the nature of NHS service providers' involvement in our study as PICs delayed by two weeks our application to the centre that would co-ordinate within Scotland and make links with England, in this case the NHS Research Scotland Coordinating Centre (NRS CC). Approvals were sought from seven Health Boards in Scotland, which were duly given over a period of 13 to 44 (median 15) working days after receipt of ethical approval. Thus, the time taken between application for ethical approval and confirming all RM&G permissions in Scotland was 75 (median 46) working days.

### R&D governance checks in England

In England the NIHR CRN CC was sent details of our study by the NRS CC 26 working days after receipt of ethical approval, although only appeared to receive the information after a further 11 working days. Due to this being the Scottish centre's first cross-border study under the new system, there was some confusion about knowing where to send the information. Unfortunately, the English coordinating centre itself did not have a process in place nor for guiding the CLRNs on PIC studies until 60 working days after our ethical approval was gained, before which the global and local checks could not be started.

In order to be able to undertake the fieldwork within the project time frame, our original deadline for receiving approvals was in June 2009, which would have allowed 70 - 80 working days after gaining ethical approval, calculated on the basis of extensive research experience as being reasonable when the original funding application for the study was submitted, peer-reviewed and granted. Due to the difficulties gaining R&D approvals, we extended this deadline to early September 2009, requiring a negotiated, no-cost, project extension from our funder.

Whilst the design of the study attempted to minimise fieldwork costs by sampling in matching areas for respondents recruited from primary and secondary care, it was not always possible to identify a willing or able lymphoma clinic, thus requiring different CLRNs to be involved. In order to secure samples for antenatal screening and caring for dementia, in England the NIHR CRN CC needed to forward information and guidance to seven CLRNs to carry out local governance checks on behalf of 14 Primary Care Trusts (PCTs). Similarly, for recruiting lymphoma patients via secondary care in England, the NIHR CRN CC needed to contact nine CLRNs in relation to four Acute Trusts and six Foundation Trusts. While Trusts are able to begin their process of deciding whether to give permission for a study, they cannot confirm this decision until they have received the Governance Report from their CLRN assuring them that the local governance checks have been completed. We only received confirmation (beyond our deadline) from one CLRN that checks were complete 169 working days after ethical approval was granted, with a warning that we could not proceed until permission was communicated from the hospital, which could take another 15 working days; i.e. 184 days in all. Given that Trusts are mandated to respond within 21 days of receiving the Governance Report, we have to assume that CLRN approvals started when the NIHR CRN CC accepted our study into the CSP record and were all finished 110 days later.

### Trust management permission in England

Notwithstanding this delay in RM&G, we had contacted all the local R&D offices to brief them on the study in the hope that this would speed up the process once global checks had been carried out. In the case of three Acute Trusts and two Foundation Trusts, permission was granted between 86 and 126 (median103) working days after ethical approval, although we only managed to secure consultants' willingness to participate in patient recruitment in three of these Trusts (one later dropped out of the study). A further two Acute Trusts gave permission well beyond our deadline. No permission was ever received from the remaining one Acute Trust and two Foundation Trusts, although it is not clear if this was due to their CLRNs failing to confirm local approval.

**Figure 2:**
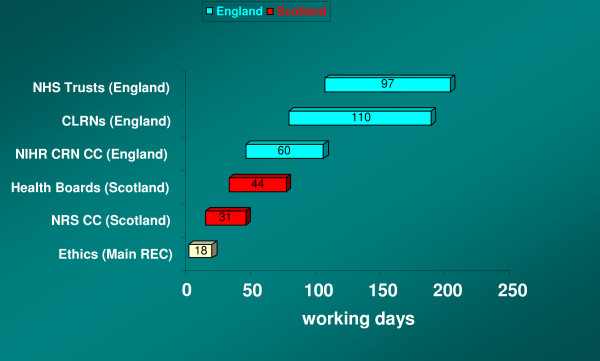
**Time taken for NHS Governance Approvals and Permission (excluding non-responses)**.

With respect to primary care, permission was received from only two of the 14 PCTs by 105 working days after ethical approval, leading us to have to abandon this part of the study, given the additional delays likely at the next stage when recruiting general practices. In total, the time between application for ethical approval and those Trust permissions that were received in England was 202 working days. The time delays in gaining approvals and permission through this governance process, excluding the CLRNs and NHS Trusts which did not respond at all, is summarised in Figure 2.

### Impact on professional recruitment

The third concurrent phase of the process of attempting to secure approval to sample from medical records involved the recruitment of professional staff. We were fortunate to benefit from the help and support of the clinical primary care and cancer research networks in Scotland and England for recruiting general practices and lymphoma clinics, respectively.

In England, in order to secure governance approvals, NIHR CRN CC advised us that we needed to provide them with details of all participating PICs before governance checks could be completed and approvals given, yet most of the Primary Care Research Networks refused to recruit general practices until governance approvals had been granted, citing as the reason that many studies do not go ahead. This caused difficulties for gaining approvals from PCTs due to the intractability of this situation. Of the two PCTs which eventually gave permission, only a handful of GPs had agreed to act as PICs by our extended deadline, leading to us having to abandon this part of the study. The cancer research network did not impose a similar requirement and we were able to recruit consultants prior to governance approvals being granted, although this was not without its difficulties.

### General issues

By the time we drew a halt on trying to obtain R&D approvals and permission and could begin to plan an accurate schedule for recruiting and interviewing individual respondents, the original schedule for the fieldwork had effectively been delayed by about 4 months, requiring considerable re-negotiation about the implementation and costs of fieldwork with the subcontractor. Not only that, but it became necessary to phase the fieldwork into three distinct periods to reflect when samples became available (Scottish lymphoma patients, followed by Scottish primary care respondents, and finally English lymphoma patients), adding further costs to the study. It is worth noting that we also had to negotiate with our funders for an extension in the study deadline of three months to achieve our revised and scaled-down objectives, without incurring any increased costs, through a reduction in expected sample sizes offsetting continuing staff costs.

In managing the project we attempted to identify the main causes of these cumulative delays, since we wished to be able to find alternative means to resolve bottlenecks and to provide information that might be required, even though everything should have been available via IRAS. Reasons for delays were rarely offered spontaneously by CLRNs or Trusts, but only as the result of enquiries made by the research team. The reasons included lack of familiarity with the new system, in particular concerning PIC studies, and apparent confusion over what was required of CLRNs and Trusts; having lost or not having received information; and waiting for information that was not required.

CLRNs and Trust R&D departments reported finding the hastily put together PIC guidance from NIHR CRN CC 'cumbersome' and reported that ours was the first study of its kind that they had been required to process using the new system. In some instances, R&D departments informed us that they had carried out all of their local checks but could not issue an approval until the CLRN had completed their four checks, which for some reason had not been completed. Other CLRNs and Trusts mistakenly thought that our study involved NHS service providers carrying out research activities, rather than acting as PICs, and so repeatedly asked for copies of non-existent documentation in relation to this.

More than once we were requested to provide information that should have filtered down directly from IRAS or the clinical research networks, which points to lack of familiarity with the IT system. In a few cases we were asked for 'enhanced' Disclosure (Scotland) or Criminal Records Bureau (England) checks, or occupational health checks, that most providers, rightly or wrongly, did not consider necessary.

In summary, from applying for ethics approval, through the minefield of RM&G approvals and recruitment of professionals, to securing respondents to our samples has taken almost a year, involving substantial additional work in progress chasing. The processes in place have led to a patchy response, which in England has resulted in just two lymphoma centres and a complete absence of primary care to sample the other two conditions, thus preventing generalisation at the national level. These problems have prevented us gaining timely RM&G approvals and permissions, which threaten the scientific integrity of the study, making it difficult to reach meaningful and robust conclusions.

## Discussion

The need for governance in health-related research, both for affirming its ethical status and for ensuring that the services have the capacity to support it, is not under dispute by the vast majority of the research community. However, there are major concerns about whether current governance arrangements are appropriate to the task and able to provide timely and genuinely supportive systems to investigators. That systems require a number of iterations before they achieve their aims is not a new or surprising phenomenon, but the current arrangements in the NHS within the UK do not yet reflect what is claimed for them.

It would appear from our case study and those quoted earlier [8,16] that there is no necessary delay in gaining ethical approval under the current framework, at least not for relatively low-risk projects using PICs, such as ours. The main time-consuming activity is in writing the extensive on-line application form, but the new IRAS system has been designed to minimise the amount of duplication of requests for information and to provide an automatic forwarding of relevant sections to downstream governance bodies.

Without a doubt the major part of the governance problem that we encountered was in the RM&G process. The newly launched NIHR CSP, while supported by a clear and reasonably efficient IRAS application process, did not initially have the necessary processes in place for dealing with all types of study, such as those involving PICs in our case. Once the protocol for PICs had been agreed the governance checks could move ahead, but it does not explain why the CLRNs still required so much time for the local checks, nor why some never responded. While CLRNs were not subject to a time target, due apparently to the number of variables involved [26], the permission from local NHS providers was required within 21 days of receiving the Governance Report. It begs the question of why so many providers never replied at all. The espoused benefit of the NIHR CSP providing a "high quality process" [1] would seem rather premature.

By contrast, the governance process in Scotland was, in our experience, a much easier and quicker system through which to gain the necessary approvals and permission. A major factor is likely to be the less fragmented nature of NHS Scotland, with its single authority structure of Health Boards, probably supported by a closer network of research support. All except one Health Board responded very quickly once the NRS CC gave the go-ahead, which was granted soon after receiving ethical approval. The rare examples of confusion about the regulations concerning PICs were relatively quickly resolved once we discussed the IRAS guidance with them. The communication difficulty between the Scottish and English co-ordinating centres slowed down the process, but may be symptomatic of the teething problems of learning a new system for cross-border studies.

Where difficulties or delays occurred, we only discovered them through repeated requests for status reports, rather than there being a sense of partnership in the process. Neither we nor our funders had any managerial control over this process, which, we would argue, requires responsibility being taken up by local management within some nationally agreed guidance.

Implementation of policy such as this is dependent upon an adequate level of resources being made available in a timely fashion, as well as a comprehensive training package for staff at the different levels, supported by transparent and accessible guidance.

The narrative we have presented in this case study offers a detailed description of the difficult journey that faces investigators attempting to sample within the NHS in England, in particular, and to a much lesser extent in Scotland. A limitation of this study is that, being a single case, it may not be generalisable to the experiences of other researchers. However, many of the problems we faced at the level of service provider were not new and, even if some of them were teething problems in the new arrangements, there are still worrying examples of lack of forethought, resources and management in the implementation stage, which will require deliberate action to resolve.

## Conclusions

Ethical approval for health-care research is carried out by independent committees, which, in attempting to protect research participants, consider not only the risks, consent procedures and data confidentiality, but also the scientific importance and research design of the study [27]. Whilst it does raise questions about why ethics committees might be duplicating the work of funding organisations in carrying out what is in large part a peer review of the research itself, part of a phenomenon known as 'ethics creep' [28], there are arguments to value this process of reflection on the process. To a large extent the lengthy application form should be relatively straightforward, given the tendering process which probably preceded it.

The recent history of gaining governance approvals and permission to approach staff or people registered on medical records has necessitated a much less bureaucratic and streamlined system for research to be established in the NHS in the UK. The new system introduced with full effect from 2009 should have some benefits over the previous system, insofar as it provides a single point of application (IRAS) through a more coherent software package, which has led to less duplication of the information that is requested. In addition there are reasonable time targets for gaining ethical approval and for NHS providers to give permission, once the up-stream clinical research network approvals have been given.

Unfortunately, the disadvantages of the current system continue to thwart the delivery of well-conducted and timely research. Unnecessary duplication still exists between the different levels of the governance framework, partly because of lack of understanding or clarity of the new regulations in place. There is a lack of over-arching time targets for the entire RM&G process, in particular relating to CLRNs in England, an apparent lack of any requirement to respond at all, either to give or withhold permission, and, as a result, the higher likelihood of studies being abandoned, or reduced in scope and depth. The consequence is a threat to the scientific contribution that research can deliver for the funds allocated.

We would support the recommendation of Appleton and colleagues [29] that a collaborative framework be set up that brings together researchers and those vested with responsibility for designing and implementing research governance processes, extending it to cover the whole of the NHS. A clear communications strategy needs to be in place to ensure all parts of the system are in harmony and understand what is required, supported by properly-financed training and IT systems. Despite the difficulties of dealing with the many variables at play, there needs to be a limit on the time taken by any part of the system, especially CLRNs, to approve or disapprove a research project taking place, and, in the case of the latter decision, with clear explanations as to why it has failed. An interesting question for the NIHR to answer would be the opportunity cost of research projects that have been abandoned or reduced in scope as a result of failures in the RM&G approvals process for reasons other than poor science.

The end results of the problems and deficiencies in the current process are inevitably poorer research outputs, with quantitative studies being underpowered and probably unrepresentative, difficulties in fulfilling the social justice agenda of giving voice to unheard population groups, lower efficiency and ultimately poorer value for money.

## Abbreviations

CLRN: Comprehensive Local Research Network; COREC: Central Office for Research Ethics Committees; GP: General Practitioner; IRAS: Integrated Research Application System; IT: Information Technology; LREC: Local Research Ethics Committee; MREC: Multi-Centre Research Ethics Committee; NHS: National Health Service; NIHR: National Institute for Health Research; NIHR CRN CC: National Institute for Health Research Clinical Research Network Coordinating Centre; NIHR CSP: National Institute for Health Research Coordinated System for gaining NHS Permission; NRES: National Research Ethics Service; NRS CC: NHS Research Scotland Coordinating Centre; PCT: Primary Care Trust; PIC: Participant Identification Centre; R&D: Research and Development; REC: Research Ethics Committee; RM&G: Research Management and Governance; SSA: Site-Specific Assessment.

## Competing interests

The authors declare that they have no competing interests.

## Authors' contributions

Both authors were actively involved in this stage of the research study that experienced the problems identified in this paper. AT conceived and designed the paper. EF managed the communications with the various NHS organisations on which the analysis was based. Both authors contributed to the writing and discussion of the various drafts, before approving the final manuscript.

## Pre-publication history

The pre-publication history for this paper can be accessed here:

http://www.biomedcentral.com/1472-6963/10/124/prepub
